# Analysis of folylpoly-γ-glutamate synthetase gene expression in human B-precursor ALL and T-lineage ALL cells

**DOI:** 10.1186/1471-2407-6-132

**Published:** 2006-05-17

**Authors:** Guy J Leclerc, Gilles M Leclerc, Ting Ting Hsieh Kinser, Julio C Barredo

**Affiliations:** 1 Department of Pediatrics and Department of Biochemistry and Molecular Biology, Medical University of South Carolina, PO Box 250558, Charleston SC, 29425, USA; 2 Department of Cell Biology and Anatomy, Medical University of South Carolina, Charleston SC, 29425, USA

## Abstract

**Background:**

Expression of folylpoly-γ-glutamate synthetase (FPGS) gene is two- to three-fold higher in B-precursor ALL (Bp- ALL) than in T-lineage ALL (T-ALL) and correlates with intracellular accumulation of methotrexate (MTX) polyglutamates and lymphoblast sensitivity to MTX. In this report, we investigated the molecular regulatory mechanisms directing FPGS gene expression in Bp-ALL and T-ALL cells.

**Methods:**

To determine FPGS transcription rate in Bp-ALL and T-ALL we used nuclear run-on assays. 5'-RACE was used to uncover potential regulatory regions involved in the lineage differences. We developed a luciferase reporter gene assay to investigate FPGS promoter/enhancer activity. To further characterize the FPGS proximal promoter, we determined the role of the putative transcription binding sites NFY and E-box on FPGS expression using luciferase reporter gene assays with substitution mutants and EMSA.

**Results:**

FPGS transcription initiation rate was 1.6-fold higher in NALM6 *vs*. CCRF-CEM cells indicating that differences in transcription rate led to the observed lineage differences in FPGS expression between Bp-ALL and T-ALL blasts. Two major transcripts encoding the mitochondrial/cytosolic and cytosolic isoforms were detected in Bp-ALL (NALM6 and REH) whereas in T-ALL (CCRF-CEM) cells only the mitochondrial/cytosolic transcript was detected. In all DNA fragments examined for promoter/enhancer activity, we measured significantly lower luciferase activity in NALM6 *vs*. CCRF-CEM cells, suggesting the need for additional yet unidentified regulatory elements in Bp-ALL. Finally, we determined that the putative transcription factor binding site NFY, but not E-box, plays a role in FPGS transcription in both Bp- and T-lineage.

**Conclusion:**

We demonstrated that the minimal FPGS promoter region previously described in CCRF-CEM is not sufficient to effectively drive FPGS transcription in NALM6 cells, suggesting that different regulatory elements are required for FPGS gene expression in Bp-cells. Our data indicate that the control of FPGS expression in human hematopoietic cells is complex and involves lineage-specific differences in regulatory elements, transcription initiation rates, and mRNA processing. Understanding the lineage-specific mechanisms of FPGS expression should lead to improved therapeutic strategies aimed at overcoming MTX resistance or inducing apoptosis in leukemic cells.

## Background

Folate antimetabolites play a central role as anticancer agents. In mammalian tissues, intracellular folates and antifolates exist as poly-γ-glutamates with typical chains ranging from five to nine residues [1-3]. Polyglutamation is catalyzed by folylpoly-γ-glutamate synthetase (FPGS) and results in increased intracellular concentration and cytoxicity of classical antifolates [4]. Furthermore, when polyglutamated, some antifolates (e.g., raltitrexed, lometrexol) increase their *Ki *against targeted enzymes by over 100-fold [5,6]. In childhood acute lymphoblastic leukemia (ALL) a strong correlation exists between FPGS expression, intracellular methotrexate (MTX) polyglutamate accumulation and treatment outcome [4,7].

The FPGS gene is controlled by at least two mechanisms: one tissue/lineage-specific and a second proliferation-dependent [8-11]. FPGS activity is distributed to both cytosolic and mitochondrial compartments of mammalian cells. In humans, these two isoforms are encoded by a single locus in chromosome region 9q (34.1–34.2) [12], and differ by the use of two alternative translational start sites within exon 1 [13]. Use of these alternative start sites translates the FPGS protein with or without the addition of a mitochondrial leader sequence. Alternative FPGS exon 1 variants (exons 1, 1A, 1B, 1C, and 2A), all spliced to exon 2, have been described [12,14]. We have demonstrated no lineage-specific differences in the expression of these alternative transcripts in human leukemia and normal tissues [15].

In mice, two promoters spaced by 10 kb were shown to express distinct functional tissue-specific FPGS isoenzymes [16]. The upstream transcript (exon A1a) was expressed only in few differentiated tissues such as liver, whereas the downstream transcript (exon 1) was expressed in dividing normal and neoplastic tissues. This dual promoter mechanism directing expression of murine isoenzymes is not conserved in humans. In contrast, the human FPGS exon 1 transcript is present in both dividing and differentiated tissues [14]. In human leukemia cells, the enzyme translated from exon 1 transcript was reported as the only catalytically active form. Transcription of the human FPGS gene appears to be controlled by a TATA-less promoter driven by a set of 8 concatameric Sp1 sites spaced within a 150 bp region upstream of exon 1 [17]. Several additional transcription factors, including NFY (Y-box) and E-box motifs have been identified within the human minimal FPGS promoter region [13,17].

Our laboratory first demonstrated that constitutive levels of FPGS mRNA, protein, and enzyme activity are two- to three-fold higher in B-precursor (Bp) ALL cells compared to T-lineage ALL [9,11]. We now report studies investigating the molecular mechanisms for this differential FPGS gene expression. Our results demonstrate that FPGS transcriptional start sites (+1) are the same in all hematopoietic lineages studied. To analyze lineage differences in FPGS promoter activity we used a FPGS promoter-luciferase gene reporter assay. All DNA fragments examined for promoter/enhancer activity exhibited higher levels of luciferase activity in CCRF-CEM *vs*. NALM6 cells. Finally, we determined the role of the putative NFY and E-box transcription factor binding sites on FPGS gene transcription using the same reporter gene assay with substitution mutants and EMSA analysis.

## Methods

### Leukemia cell lines

The human leukemia cell lines CCRF-CEM (T-ALL) and REH (Bp-ALL t(12;21)) were obtained from the American Type Culture Collection. NALM6 (Bp-ALL) cell line was obtained from DSMZ (Germany). RCH-ACV (Bp- ALL t(1;19)) was kindly provided by Dr. Stephen Hunger (UFL, Gainesville, FL). All cell lines were grown in RPMI 1640 (Sigma) supplemented with 10% FBS at 37°C and 5% CO_2_. Normal bone marrow (BM) was extracted from normal volunteers. We obtained institutional review board approval, and IRB approved informed consent was obtained from normal volunteers prior to participation.

### RNA isolation and real-time RT-PCR

Total RNA was isolated using the RNeasy kit (Qiagen Inc). FPGS exons 14 and 15 were RT-PCR amplified [15] and quantitated using pFPGS-cDNA, a pCR2.1-TOPO plasmid containing the human FPGS cDNA gene (1926 bp; Table 1). Briefly, the 1926 pb FPGS cDNA gene was amplified by PCR using primers 1exonF and 15exonR-c1934 (Table 1) from total RNA reverse transcribed with primer 15exonR-c2107 (5'-GGCCAGGCAGCGCACACAAT). Results were normalized to β-actin mRNA expression. All real-time PCR reactions (SYBR green) were performed using the BIO-RAD iCycler iQ system (Bio-Rad) [18].

### Nuclear run-on assay

Nuclear run-on assay was performed as described by Hanson et al. [19]. One μg of FPGS and 18S RNA cDNAs were blotted, UV cross-linked and hybridized with nascent labeled RNA transcripts. After washing, membranes were exposed to XAR-5 film (Kodak). Densitometric scan analysis was performed using the GelPro Analyzer program. Results were normalized to the level of 18S RNA and statistic achieved using one-tailed, Student paired *t*-test (GraphPad Prism, version 2.01). All data are expressed as mean ± S.E.M.

### 5'-Rapid amplification of cDNA ends (RACE)

Amplification of the 5'-termini of FPGS mRNA was performed using the 5'-RACE system Version 2.0 (Invitrogen Corporation). Briefly, polyA+ RNA isolated from cells using the Oligotex Direct mRNA mini kit (Qiagen) were reverse transcribed using the oligonucleotide Exon5R-F8 (5'-CTTGGTGAAGAGCTCAGGACTG), a gene-specific primer in exon 5. The poly dC-tailed cDNA products were amplified using a nested primer in exon 2, Exon2R-F4 (5'- ACTCCGTGCCAGGTACAGTTCCATG), and the abridged anchor primer. Primary PCR products were re-amplified using an exon 2 upstream nested primer, 2exonR-2381 (5'-CAGGTAGCCGGCATTGGTCTG), and the abridged universal amplification primer. 5'-RACE products were separated on a 2.5% agarose gel, purified and cloned into the pCR2.1-TOPO vector (Invitrogen). Identity of the 5'-RACE products was determined by nucleotide sequence.

### Construction of FPGS-luciferase reporter gene fusions

Regions of the FPGS gene promoter were generated by PCR, cloned into pCR2.1-TOPO vector and sub-cloned into pGL3-basic vector (Promega) or pGL2628-ATGm/c. Plasmids and primers used in this study are listed in Table 1. The BAC clone RCPI-11 465E22 contains 188,098 bp of human chromosome 9 including 104,600 bp upstream of FPGS exon 1 (obtained from Dr. P.J. de Jong, Children's Hospital Oakland). PCR conditions were optimized for each of these fragments.

### Nucleofection and luciferase reporter gene assays

The CCRF-CEM, NALM6 and REH cell lines were transfected by nucleofection (Amaxa Biosystems) following manufacturer's protocol. Briefly, 5 × 10^6 ^cells were resuspended in 100 μl of solution V^® ^(CCRF-CEM and NALM6) or R^® ^(REH) and mixed with 2.5 μg of plasmid pGL1374 (FPGS promoter::luc) or equimolar concentration of other FPGS promoter::luc plasmids, and 3 μg of pCMVβ. Cells were incubated at 37°C/5% CO_2 _for 24 hrs, harvested, washed twice with cold PBS 1X and resuspended in dual-light lysis buffer to yield cellular extracts. Luciferase and β-galactosidase activities were assayed using the dual-light reporter gene assay system (Tropix, Inc.). Transfection efficiencies and cell viability were monitored by flow cytometry. Cell viability was 70–75% and 78–85% for CCRF-CEM and NALM6 cells, respectively. Statistical tests were achieved using one-tailed, Student paired *t*-test (GraphPad Prism, version 2.01).

### Methylation-specific PCR analysis

Genomic DNA was isolated from CCRF-CEM and NALM6 cells using the DNeasy kit (Qiagen, Inc.) and treated with sodium bisulfite to convert unmethylated cytosine to uracil residues while 5-methylcytosines remain unaltered (CpGenome DNA modification kit [Chemicon International]). Identification of CpG islands and design primer sets were determined using the MethPrimer program [33]. Chemically converted genomic DNA was PCR amplified using specific primer sets for methylated and unmethylated forms (Table 1). Methyl-specific PCR (MSP) products were resolved on a 3% agarose gel.

### Site-directed mutagenesis

The QuikChange II XL Site-Directed Mutagenesis Kit (Stratagene) was used to generate substitution mutants of the human FPGS mitochondrial and cytosolic initiation codons (ATG) from the pGL2628 plasmid using oligonucleotides QC19934F/QC19934R and QC20060F/QC20060R, respectively (Table 1). Similarly, FPGS putative NFY868 and E-box952 binding sites were mutagenized using the plasmid pGL2628-ATGm/c plasmid DNA template. Primers NFY-868F/NFY-868R and E47-952F/E47-952R were used for the NFY and E-box mutagenesis, respectively (Table 1). Mutations were confirmed by nucleotide sequencing.

### Nuclear extracts and electrophoretic mobility shift assay (EMSA)

Nuclear extracts were prepared from CCRF-CEM and NALM6 cells using the NE-PER Nuclear and Cytoplasmic Extraction Reagents (Pierce, Biotechnology, Inc.). DNA-protein interactions were carried out and detected using the LightShift Chemiluminescent EMSA kit (Pierce, Biotechnology, Inc.). Each EMSA reactions contained 25 nM labeled FPGS -32/-14 (5'-CTGCGCTGATTGGCTGGGG) oligonucleotides, 50 ng Poly (dI-dC) and 10 μg of nuclear protein. When required, competitive unlabeled NFY DNA oligomer, unlabeled Epstein-Barr nuclear antigen (EBNA) DNA, and NFY antibody (CBF-A (C20) or CBF-B (H209) (Santa Cruz, Biotechnology, Inc.)) were added to EMSA. The DNA-protein complexes were resolved on non-denaturing 5% TBE polyacrylamide gels (Bio-Rad).

## Results

### FPGS transcription rate and mRNA transcription start sites in Bp- and T-ALL

**Figure 1:**
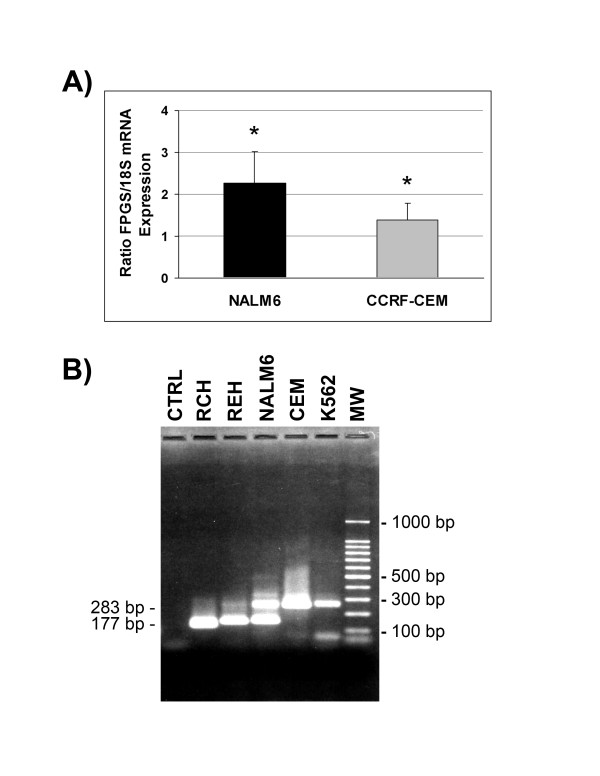
**FPGS transcription initiation rate and start sites in CCRF-CEM and NALM6 cells**. **(A) **Nuclear run-on assays. Intact nuclei were isolated from CCRF-CEM and NALM6 cells, and nascent RNA transcripts were labeled with [α-^32^P]UTP, purified, and hybridized to 1 μg of FPGS and 18S cDNAs blotted on nitrocellulose membrane. Integration densitometry values of FPGS and 18S signals were determined using the Gel-Pro program. Bars represent the mean of four independent experiments ± S.E.M. Comparison of transcription rates were achieved using a Student's *t*-test (*, *p *< 0.05). **(B) **5'-RACE products of FPGS mRNA from RCH (RCH-ACV), REH, NALM6, CEM (CCRF-CEM) and K562 cells are indicated. The lanes "CTRL" and "MW" represent negative control and the 100 bp DNA ladder, respectively. See Materials and Methods for details.

To investigate whether differences in FPGS mRNA expression in Bp-ALL and T-ALL resulted from differences in FPGS promoter transcription rate, we determined the frequency of transcription initiation in CCRF-CEM and NALM6 cells using nuclear run-on assays. As shown in Figure 1A, ratio of FPGS/18S mRNA transcription rate was 1.64-fold higher in NALM6 (2.26 ± 0.768) *vs*. CCRF-CEM (1.37 ± 0.416) cells (*p *< 0.05). These results are consistent with the observed two- to three-fold higher levels of FPGS mRNA, protein expression and activity in NALM6 *vs*. CCRF-CEM [9,11,15].

To uncover potential regulatory regions involved in the lineage differences in expression of the FPGS gene, we localized the FPGS promoter in Bp-ALL *vs*. T-ALL cell lines by mapping FPGS transcription initiation sites using 5'-RACE. These experiments detected a long (~280 bp) and a short (~180 bp) fragment, which were individually characterized by nucleotide sequence analysis to encode mitochondrial/cytosolic and cytosolic FPGS, respectively. Long fragments were detected in CCRF-CEM (T-ALL) whereas both short and long fragments were amplified from NALM6 and REH (Bp-ALL) (Figure 1B). Therefore, both Bp-ALL and T-ALL cells use the same promoter to transcribe FPGS mRNA but lineage differences in mRNA transcripts exist.

### FPGS gene promoter activity in Bp- and T-ALL lineages

**Figure 2:**
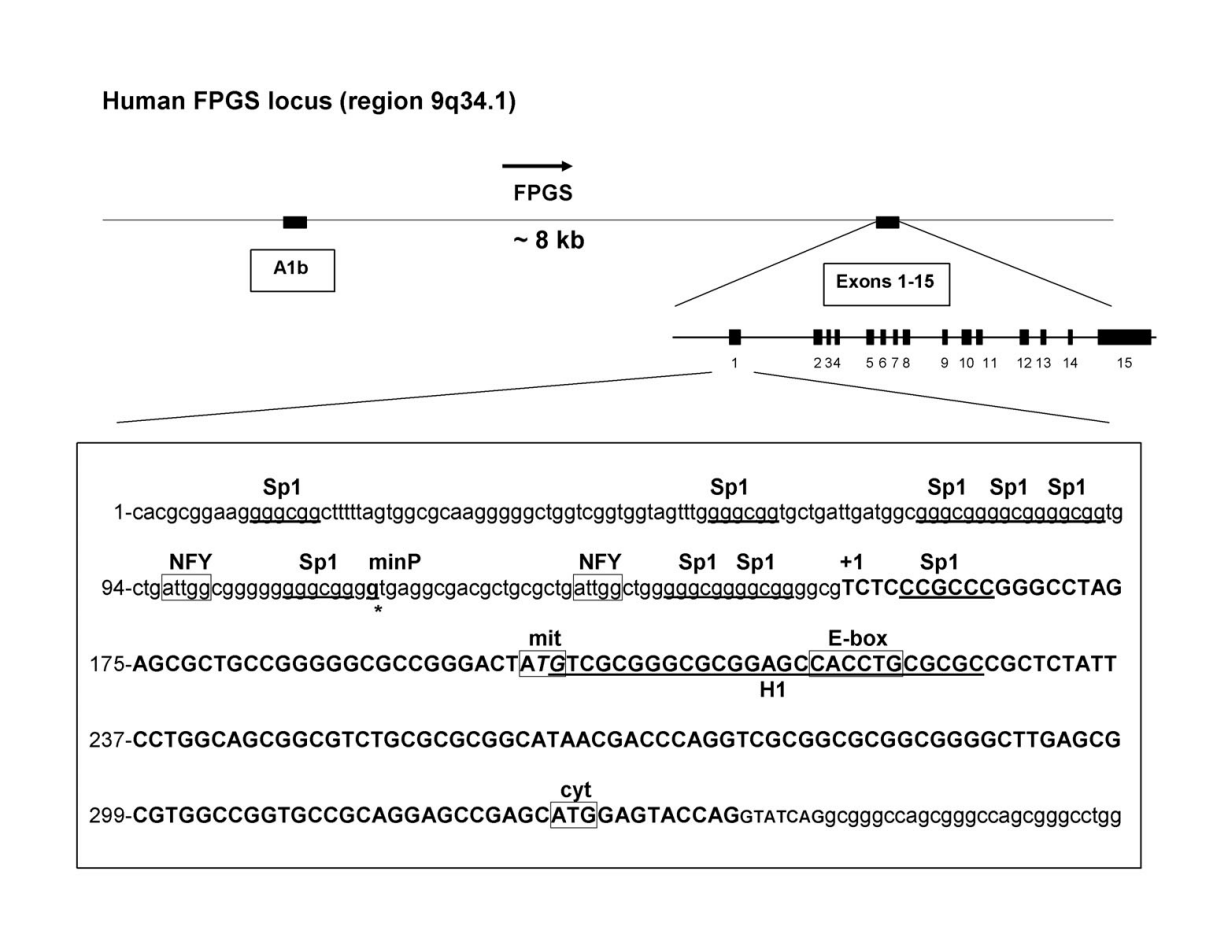
**Schematic representation of the human FPGS gene and promoter region**. FPGS exon A1b, exons 1 to 15, and the nucleotide sequence of the FPGS gene promoter region (accession # AL162586) are depicted. Putative transcription factor recognition motifs were identified using the MatInspector program from Genomatix (Release 7.3.1) [34]. Sp1 (GGGCGG; +5, -10, -15, -65, -72, -77, -82, -102, -147), NFY-box (CCAAT; -20 and -57), and E-box (CANNTG; +61) transcription factor binding sites are indicated. The protected DNase I footprint region, called H1 (+45 to +71) is shown underlined [17].

**Figure 3:**
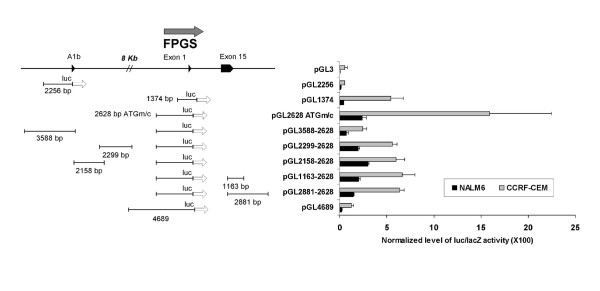
**FPGS promoter-luciferase constructs and activity in CCRF-CEM and NALM6 cells**. Genetic organization of the FPGS promoter-luciferase constructs is shown with their normalized luciferase/β-galactosidase activity in CCRF-CEM and NALM6 cells. Horizontal lines represent fragments cloned into pGL3 (luc) vector. Size of the fragments is indicated. Pointed boxes represent exons, and arrows direction of gene expression. Exponential cultures of CCRF-CEM and NALM6 cells (5 × 10^6^) were transfected by nucleofection with equimolar concentration of pGL1374/pCMVβ and other FPGS promoter::luc constructs as described in Materials and Methods. Luciferase activity (RLU; relative light units) was initially subtracted from the level of luciferase activity detected with pGL3 (empty vector) and normalized with respect to the level of β-galactosidase activity (RLU). Each experiment was performed at least two times in triplicate. Bars represent mean ± SD. See details in Materials and methods.

The human FPGS minimal promoter has been characterized in CCRF-CEM cells and encompassed a region starting -43 bp from the main transcriptional start site to +150 bp of exon 1 [17] (see Figure 2). To further investigate the mechanisms that control FPGS transcription in human hematopoietic cells, we analyzed and compared DNA fragments located upstream of exon A1b and in the 5'-flanking region of exon 1 (encompassing the previously described minimal promoter region) for promoter/enhancer transcriptional activity in CCRF-CEM and NALM6 cells. FPGS-luciferase transcriptional gene fusions were constructed and assayed as described in Material and Methods. Under our experimental conditions, transfection efficiencies were 38–50% and 42–50% in CCRF-CEM and NALM6 cells, respectively. As shown in Figure 3, DNA fragments located upstream of exon A1b (pGL2256 and pGL3588-2628) yielded no promoter or enhancer activity in both cell lines suggesting that the 5'-flanking region of exon A1b exerts no regulatory activity on human FPGS expression. The higher level of luciferase activity detected in pGL3588-2628 *vs*. pGL2256 is likely the result of the presence of the additional fragment of 2628 bp contained in pGL3588-2628 that included the FPGS minimal promoter region. When DNA fragments from the 5'-flanking region of exon 1 (pGL1374, pGL2628-ATGm/c, pGL4689) encompassing the described minimal promoter were analyzed, we found 4- to 12-fold higher level of luciferase/β-galactosidase activity in CCRF-CEM compared to NALM6 cells (Figure 3). To validate our reporter gene assay and rule out any technical differences in luciferase activity in NALM6 *vs*. CCRF-CEM cells, we assayed a CMV promoter-luciferase driven vector (pCMV-luc) and found no significant differences in both cell lines (data not shown). Therefore, these data indicate that DNA fragments containing the previously described minimal promoter region are not sufficient to effectively drive FPGS transcription in Bp-ALL (NALM6) compared to T-ALL (CCRF-CEM). Similar experiments with other Bp-ALL cell lines such as RCH-ACV (t(1:19)) and REH (t(12;21)) were performed and yielded same results (data not shown). To exclude that differences in nucleotide sequence were responsible for low promoter activity observed in Bp-ALL, we PCR amplified and sequenced the 1374 bp fragment containing the minimal promoter from normal bone marrow (BM), CCRF-CEM and NALM6 cells. No difference in nucleotide sequence was detected.

The inability of the minimal promoter region to efficiently drive FPGS transcription in NALM6 cells implied that additional unidentified regulatory regions may be required in Bp-ALL cells. To test this hypothesis we then investigated the presence of enhancers or transcription factors required for FPGS transcription in NALM6 *vs*. CCRF-CEM cells, by analyzing the effect in *cis *of DNA fragments located within the exons A1b-1 and in the 3'-UTR of the FPGS gene on the FPGS-luc expression of pGL2628-ATGm/c. As shown in Figure 3, plasmids pGL2299-2628, pGL2158-2628, pGL1163-2628, and pGL2881-2628 constructs yielded 1.8- to 4.2-fold lower level of luciferase activity in NALM6 *vs*. CCRF-CEM cells. Therefore, the regulatory elements required for FPGS gene expression in Bp-ALL appear not to be localized within a region encompassing 12 kb upstream of exon 1 nor within the 3'-untranslated region.

### Methylation status of the FPGS promoter in Bp- and T-lineage

**Figure 4:**
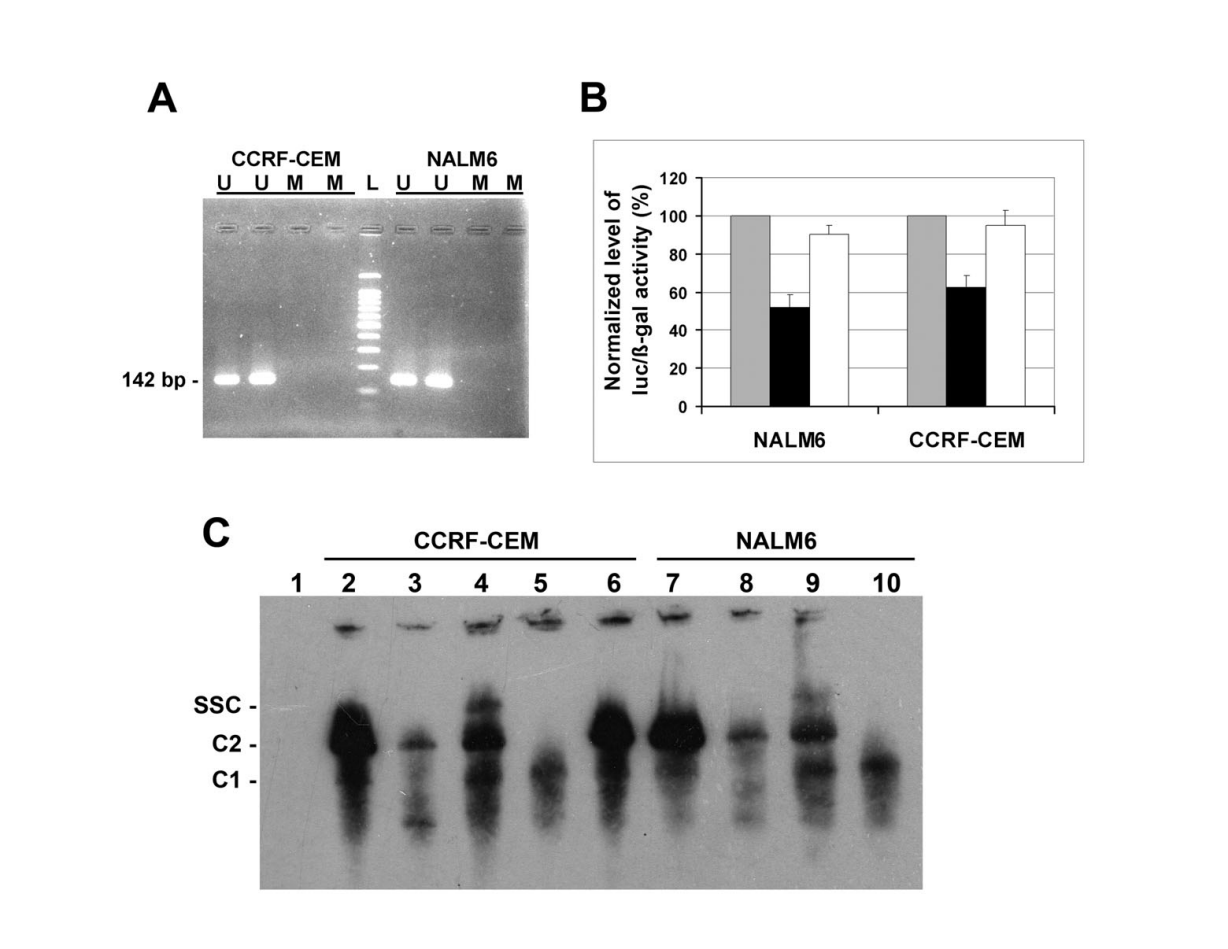
**Methylation status and analysis of NFY and E-box transcription binding sites on FPGS expression**. **(A) **Genomic DNA from CCRF-CEM and NALM6 cells was treated with sodium bisulfite and DNA region of the FPGS gene promoter was PCR-amplified using primer sets specific to methylated (M) and unmethylated (U) forms (Table 1). Expected MSP products were M, 139 bp and U, 142 bp. L, 100 bp ladder molecular weight marker. **(B) **The putative NFY and E-box transcriptional binding sites were mutagenized as described in Materials and Methods. Wild type pGL2628-ATGm/c (grey bars) and mutated pGL868NFY-ATGm/c (black) and pGL952Ebox-ATGm/c (white) plasmids were transfected by nucleofection in CCRF-CEM and NALM6 cells. Level of luciferase activity (RLU) was initially subtracted from the level of luciferase activity detected with pGL3 (empty vector), standardized with respect to the level of β-galactosidase activity, and normalized to wild type pGL2628-ATGm/c level. Each experiment was performed at least two times in triplicate. Data are expressed as mean ± S.E.M. **(C) **EMSA of biotinylated double stranded oligonucleotides containing the FPGS NFY binding site (region -32/-14) incubated with nuclear extracts prepared from CCRF-CEM (lanes 2–6) or NALM6 (lanes 7–10) cells. For competition experiments, 0.4 μM of unlabeled NFY (lanes 3 and 8) or EBNA oligonucleotides (lane 6) were included in the reaction mixture. NFY antibody CBF-A (lanes 4 and 9) or CBF-B (lanes 5 and 10) were added to the reaction mixtures. Specific DNA-protein complexes C1 and C2, and the supershifted complex (SSC) are indicated. Lane 1 represents negative control with no nuclear extract.

DNA methylation has been associated with transcriptional inactivation and gene silencing [20,21]. Nucleotide sequence analysis of the 1374 bp fragment (-791 to +582) containing the FPGS minimal promoter predicted one CpG island (54% GC content) at position -330 to +294. We determined whether DNA methylation could contribute to the lineage-specific differences in FPGS expression in ALL cell lines by methylation-specific PCR (MSP) analysis using bisulfite-treated DNA. Primers sets were designed to anneal to unmethylated DNA (U) and methylated templates (M). As shown in Figure 4A, amplification products (142 bp) were detected in both CCRF-CEM and NALM6 bisulfite-treated nuclear DNA with unmethylated primers (U) indicating a preferentially unmethylated CpG island in both cell lines. Control experiments with untreated DNA or absence of template yielded no products (data not shown). Quantitative analysis of cytosine methylation was determined in both cell lines by sequencing of the 142 bp unmethylated PCR amplicon. This analysis revealed identical number of unmethylated cytosines in both cell lines. Therefore, DNA methylation of the CpG island region which contains the described FPGS minimal promoter does not play a role in the observed lineage-specific differences in FPGS expression in ALL cell lines.

### Role of putative NFY-box and E-box binding sites on FPGS expression

To identify specific regulatory elements involved in FPGS gene transcription, we analyzed the nucleotide sequence of the minimal FPGS gene promoter for presence of known transcription factor recognition motifs using the MatInspector program (Genomatix, release 7.3.1). As shown in Figure 2, two Sp1 (GGGCGG; -10, -15), one reverse Sp1 (+5), one inverted NFY-box (CCAAT; -20), and one E-box (CANNTG; +61) transcription factor binding sites were identified within the FPGS minimal promoter (minP). We examined the role of putative NFY and E-box DNA binding sites by generating mutants at each site with substitutions reported to reduce or abolish gene promoter activity [22,23]. Mutant constructs for NFY (NFY-868; CCAAT → C**TTT**T) and E-box (Ebox-952; CACCTG → CA**TTC**G) were co-transfected with pCMVβ in both CCRF-CEM and NALM6 cells and assayed for luciferase and β-galactosidase activities. As shown in Figure 4B, mutation in the NFY site (NFY-868) reduced the level of FPGS transcription by 45% in NALM6 (p < 0.001) and 40% in CCRF-CEM cells (p < 0.005) when compared to the wild type construct (pGL2628-ATGm/c). Normalized luciferase activities were 1.9 × 10^-2 ^± 2.0 × 10^-3 ^(pGL2628-ATGm/c) and 1.0 × 10^-2 ^± 1.2 × 10^-3 ^(pGL868NFY-ATGm/c) in NALM6, and 2.4 × 10^-1 ^± 2.4 × 10^-2 ^(pGL2628-ATGm/c) and 1.5 × 10^-1 ^± 1.4 × 10^-2 ^(pGL868NFY-ATGm/c) in CCRF-CEM cells. These data suggest that the NFY binding site is required to activate FPGS gene transcription in lymphoid cells. In contrast, no significant differences were observed in either cell line with the mutant Ebox-952 construct, suggesting a negligible role in FPGS expression (Figure 4B). In NALM6 and CCRF-CEM cells, the normalized level of luciferase activity was 1.6 × 10^-2 ^± 8.4 × 10^-4 ^(pGL952Ebox-ATGm/c) and 2.2 × 10^-1 ^± 1.8 × 10^-2 ^(pGL952Ebox-ATGm/c), respectively.

To establish NFY protein interaction with the NFY-box element (CCAAT) at position -20 upstream the FPGS gene promoter, we performed electrophoretic mobility shift assays (EMSAs) using an oligonucleotide probe containing FPGS gene sequence from -32 to -14. The sequences of double-stranded biotinylated oligonucleotide probes and competitors were incubated with nuclear extracts prepared from CCRF-CEM or NALM6 cells. The EMSA revealed the formation of two complexes with the NFY oligonucleotide probe (Figure 4C, C1 and C2). To further ascertain the specificity of binding, we demonstrated that the formation of complexes C1 and C2 were inhibited by excess amounts of unlabeled NFY oligonucleotides (Figure 4C, lanes 3 and 8) but not with unlabeled non-specific Epstein-Barr nuclear antigen (EBNA) oligonucleotides (Figure 4C, lane 6). To directly evaluate the proposed participation of NFY in the shifted complexes, anti-NFY peptide (CBF-A and CBF-B subunits) antibodies were incubated with EMSA binding reactions before electrophoresis. Addition of anti NFY-A (CBF-A) antibody to these reactions resulted in strongly retarded supershift complex (SSC) (Figure 4C, lanes 4 and 9). In contrast, addition of NFY-B (CBF-B) antibody abolished the formation of the complex C2 (Figure 4C, lanes 5 and 10) without formation of a supershifted band. These data clearly demonstrate that NFY transcription factor is present in the complex that binds to the putative NFY binding site (CCAAT) at position -20.

## Discussion

Folate antimetabolites such as MTX are essential chemotherapeutic drugs in the treatment of children with acute lymphoblastic leukemia (ALL). MTX is retained within the cell by cellular metabolism catalyzed by the enzyme FPGS. The human FPGS promoter has been previously characterized using CCRF-CEM cells and it encompasses a GC-rich region without a typical TATA sequence, usually a characteristic of housekeeping genes and proto-oncogenes. The minimal portion of the promoter required to drive transcription in CCRF-CEM cells consists of a region starting -43 to +150 of the main transcription start site including part of exon 1 [17]. When 5'-flanking sequences upstream of exon 1 were analyzed for promoter and/or enhancer activity in Bp- *vs*. T-cells, we found significantly lower luciferase activity in the Bp-ALL cell line NALM6 compared to the T-ALL cell line CCRF-CEM. These results are opposite of what one would predict based on the known lineage differences resulting in two- to three-fold higher FPGS expression Bp- *vs*. T-ALL [9,11]. These low levels of FPGS-luciferase activity observed with our constructs in NALM6 *vs*. CCRF-CEM cells, lead us to conclude that the minimal promoter region is not sufficient to effectively drive FPGS transcription in Bp-cells (NALM6). Consequently, we hypothesize that additional regulatory elements are required to drive FPGS expression in Bp-ALL. Our analysis failed to demonstrate promoter and/or enhancer activity in the immediate 5'-flanking region of exon A1b, between exons A1b and 1, and in the 3'-UTR. We propose that Bp-cell specific enhancer(s) are required for FPGS gene expression in Bp-ALL cells and that distant yet unidentified regulatory loci exist.

The human FPGS transcription start sites have been mapped previously in human CCRF-CEM and HepG2 (hepatoma) cells and shown that FPGS transcription was initiated from multiple start sites spread over 80 bp clustered in two major regions differing by the presence of the mitochondrial *vs*. cytosolic initiation codons [13,14,17]. The first transcription initiation site generates a long transcript encoding both the mitochondrial and cytosolic isoforms of the enzyme, while the second start site generates a shorter transcript encoding only the cytosolic protein. Under our conditions, these two major transcripts were detected only in Bp-ALL (NALM6 and REH) whereas in T-ALL (CCRF-CEM) cells only the longer transcript was detected. NALM6 cells which exhibited higher level of FPGS mRNA expressed both transcripts, while in REH and RCH-ACV which expressed intermediate levels of FPGS mRNA the longer transcript (mitochondrial/cytosolic) was either faint or absent, and in CCRF-CEM cells expressing the lowest level of FPGS mRNA the shorter transcript (cytosolic) was absent. This finding is consistent with and underscores regulatory differences in the expression of the FPGS gene between Bp- and T-lineage ALL cells. It has also led us to hypothesize that the additional expression of the shorter transcript encoding for additional cytosolic protein in NALM6 cells could contribute to higher FPGS mRNA expression leading to higher cytosolic protein expression in these cells. Although speculative at this time, this hypothesis which is currently being tested in our laboratory is consistent with the higher protein and enzymatic activity observed in Bp-ALL *vs*. T-ALL. If confirmed, the responsible regulatory elements could be used as targets for molecular or pharmacological interventions aimed at increasing FPGS expression and overcome *de novo *or acquired resistance in selected leukemic phenotypes were low FPGS expression mediates unresponsiveness to MTX. An alternative, although unlikely explanation is that failure from our 5'-RACE conditions to amplify the short transcript or enriched amplification of the longer form in CCRF-CEM cells resulted in these findings.

The 5'-flanking region of the downstream FPGS promoter contains eight forward/one reverse Sp1, two inverted NFY-boxes, and one E-box putative binding sites (see Figure 2) in which one NFY, one E-box, and three Sp1 binding sites are present within the FPGS minimal promoter region [17]. Functional activity of these Sp1 sites was previously demonstrated [17]. Herein, we determined that the putative transcription factor binding site NFY, but not E-box, plays a positive role in FPGS transcription in both Bp- and T-lineages. Several studies have demonstrated that the CCAAT box plays a role in gene transcription by binding specific transcription factors such as C/EBP, NF-1, and NFY [24-26]. EMSA experiments confirmed that the NFY transcription factor is part of the complex that binds the CCAAT sequence within the FPGS promoter. The NFY transcription factor is involved in the regulation of many TATA-less promoter genes such as RAG-1, [22,27], EPHX1 [25], and Gfi-1B [28]. Therefore, it is not unexpected that NFY plays a role in the regulation of the TATA-less FPGS promoter. In addition, it has been shown that NFY cooperates with many adjacent transcription factors such as GATA-1 [28,29], C/EBPα [25], and Sp1 [30], through protein-protein interactions to mediate gene transcription. Ongoing studies are investigating the interactions of NFY and some of these proteins as regulators of FPGS gene expression.

Within the FPGS promoter region we identified one CpG island at position -330 to +294 encompassing Sp1 and NFY sites. We found no role of promoter specific methylation in differential FPGS gene expression between CCRF-CEM and NALM6 cells. Promoter regions with adjacent CpG islands were found to initiate multiple transcripts similar to those identified in FPGS transcription start sites [31]. Therefore, nuclear factor(s) such as Sp1 that binds to GC-rich motifs within the CpG island and NFY could operate in conjunction to regulate human FPGS gene transcription.

## Conclusion

We demonstrated that the minimal FPGS promoter region previously described in CCRF-CEM is not sufficient to effectively drive FPGS transcription in NALM6 cells, suggesting that different regulatory elements are required for FPGS gene expression in Bp-cells. Our 5'-RACE analysis showed that two major transcripts encoding the mitochondrial/cytosolic and cytosolic isoforms were expressed in Bp-ALL (NALM6 and REH) whereas T-ALL (CCRF-CEM) cells expressed only the mitochondrial/cytosolic transcript. We determined that the putative transcription factors binding site NFY, but not E-box, plays a role in FPGS transcription in both Bp- and T-lineages. Taken together, our data indicate that the control of FPGS expression in human hematopoietic cells is complex and involves lineage-specific differences in regulatory elements, transcription initiation rates, and mRNA processing. Understanding the lineage-specific mechanisms of FPGS expression should lead to improved therapeutic strategies aimed at overcoming MTX resistance in leukemic cells by upregulating FPGS and increasing accumulation of MTX-PGs in those phenotypes with low FPGS expression. Physiologically FPGS is also known to be essential for eukaryotic cell survival although its targeted inhibition as a novel anticancer strategy has been elusive [32]. Further understanding of the genetic mechanisms that control FPGS expression could also lead to the development of novel molecular strategies capable of inducing apoptosis in leukemic cells by selectively turning off FPGS expression and could translate to better treatment outcomes for children with high-risk or refractory ALL.

## Competing interests

The author(s) declare that they have no competing interests.

## Authors' contributions

GJL conceived of the study, participated in its design, carried out the molecular cloning, 5'-RACE, site-directed mutagenesis, EMSA, methylation-specific PCR, transfection and luciferase/β-galactosidase assays, and drafted the manuscript. GML participated in the primers design, site-directed mutagenesis studies, EMSA, and statistical analysis. TTHS participated in the nuclear run-on assays. JCB conceived of the study, participated in its design and coordination, and drafted the manuscript. All authors read and approved the final manuscript.

## Pre-publication history

The pre-publication history for this paper can be accessed here:



## Figures and Tables

**Table 1:** Oligonucleotides and plasmids used in this study.

PCR products/Plasmids	Forward and Reverse primers/Description
1163 bp	exon15-53F (5'-CGTTCTCCCCATGAACTTACA)
	exon15-1215R (5'-AGGGTCTAGGCTGCAAGAAAG)
1374 bp	intron0F-141 (5'-ACCCCAGCAGTGTGTGAAGAG)
	intron1R-6537 (5'-CCCACCCCCAGATCAATACTC)
1926 bp	1exonF (5'-GGGGGCGCCGGGACTATGTCG)
	15exonR-c1934 (5'-TCTCCCGGCCTCCCATCCCAA)
2158 bp	46714F (5'-AGAAGTTCTGGTGGGAAGGAA)
	48871R (5'-CAGACCCTGGACTAGATGCTG)
2299 bp	48538F (5'-TAAGTGGGGGATGAGTCCAG)
	50836R (5'-CCCAGATTCCAGCATCCTAA)
2628 bp	17595F (5'-GGCGCAGAGGCTAAGAATAAG)
	20202R2 (5'-CCCCCAGCCTCCAGGATGTTC)
2881 bp	exon15-53F (5'-CGTTCTCCCCATGAACTTACA)
	exon15-2933R (5'-CCACACAGGAGTCAGGAATGT)
3588 bp	F8256K (5'-GAGAGAGGTACCCCCGTGACTCCTGGTGGCTGC)
	R11843K (5'-AATTCGGTACCCCTGGTCACCGGGTTCTCCTA)
4709 bp	UA1F1979 (5'-CGCACACACCGCCAACTGTTC)
	UA1R6667 (5'-CCTGGTCACCGGGTTCTCCTA)
4970 bp	UAP-15639 (5'-GAGTGGCCCTTATGTACCGAC)
	1R-20591 (5'-GGGCAACCGGCTCTTGAC)
ATGc	QC20060F (5'-CGCAGGAGCCGAGC***TC***GGAGTACCAGGTAT)
	QC20060R (5'-ATACCTGGTACTCC***GA***GCTCGGCTCCTGCG)
ATGm	QC19934F (5'-GCGCCGGGACTA***GT***TCGCGGGCGCGG)
	QC19934R (5'-CCGCGCCCGCGA***AC***TAGTCCCGGCGC)
NFY-868	NFY-868F (5'-GACGCTGCGCTGA***AAA***GCTGGGGGCGGGGC)
	NFY-868R (5'-GCCCCGCCCCCAGC***TTT***TCAGCGCAGCGTC)
Ebox-952	E47-952F (5'-GGGCGCGGAGCCA***TTC***GCGCGCCGCTCTATTC)
	E47-952R (5'GAATAGAGCGGCGCGC***GAA***TGGCTCCGCGCCC)
139 bp (MSP)^2^	M19571F 5'-GTAGAGATTGAGGGTTGTTGATTC
	M19709R 5'-CGAAACCAAATTTATAAATATACGCT
142 bp (MSP)^2^	U19571F 5'-GTAGAGATTGAGGGTTGTTGATTT
	U19712R 5'-CCTCAAAACCAAATTTATAAATATCACT
pCR1163	1163 bp PCR fragment from NALM6 cloned into pCR2.1 TOPO
pCR1374	1374 bp PCR fragment from BM cloned into pCR2.1-TOPO
pCR2158	2158 bp PCR fragment from NALM6 cloned into pCR2.1-TOPO
pCR2299	2299 bp PCR fragment from NALM6 cloned into pCR2.1-TOPO
pCR2628	2628 bp PCR fragment from BAC RCPI-11 cloned into pCR2.1-TOPO
pCR2881	2881 bp PCR fragment from NALM6 cloned into pCR2.1-TOPO
pCR4.7A1b	4709 bp PCR fragment from BM cloned in pCR2.1-TOPO
pCR4970	4970 bp PCR fragment from BAC RCPI-11 cloned into pCR2.1-TOPO
pFPGS-cDNA	1926 bp PCR from NALM6 cloned into pCR2.1-TOPO
pGL1163-2628	1163 bp *Acc*65I fragment PCR from pCR1163 cloned in pGL2628ATGm/c
pGL1374	1374 bp *Hin*dIII PCR fragment from pCR1374 cloned into pGL3
pGL2158-2628	2158 bp *Acc*65I PCR fragment from pCR2158 cloned in pGL2628ATGm/c
pGL2256	2316 bp *Hin*dIII fragment from pCR4.7-A1b cloned into pGL3
pGL2299-2628	2299 bp *Acc*65I PCR fragment from pCR2299 cloned in pGL2628ATGm/c
pGL2628	2628 bp *Sac*I-*Eco*RV fragment from pCR2628 cloned into pGL3
pGL2628-ATGc	cytosolic ATG mutagenized to TCG (ATGc) in pGL2628
pGL2628-ATG	mitochondrial ATG mutagenized to AGT (ATGm) in pGL2628
pGL2628-ATGm/c	cytosolic ATG mutagenized to TCG (ATGc) in pGL2628-ATGm
pGL2881-2628	2881 bp *Acc*65I PCR fragment from pCR2881 cloned in pGL2628ATGm/c
pGL3588-2628	3588 bp *Acc*65I PCR fragment from pCR4.7A1b in pGL2628ATGm/c
pGL4689	4708 bp *Eco*RV-*Bam*HI fragment from pCR4970 cloned into pGL3
pGL868NFY-ATGm/c	NFY-868 mutation in pGL2628-ATGm/c
pGL952NFY-ATGm/c	Ebox-952 mutation in pGL2628-ATGm/c

^1^Nucleotides substituted are in bold italic. ^2 ^MSP, Methyl-Specific PCR BM, normal human bone marrow genomic DNA.

To construct pGL1163-2628, pGL2158-2628, pGL2299-2628, pGL2881-2628, and pGL3588-2628, a sequence (5'-AATTCGGTACC) containing the *Acc*65I restriction site (underlined) was added to the 5'-end of the oligonucleotide primers used for the PCR amplification.
